# A randomized multicenter evaluation of the efficacy of 0.15% hyaluronic acid versus 0.05% cyclosporine A in dry eye syndrome

**DOI:** 10.1038/s41598-022-21330-0

**Published:** 2022-11-04

**Authors:** Ji Eun Lee, Sangyoon Kim, Hyung Keun Lee, Tae-Young Chung, Jae Yong Kim, Chul Young Choi, So Hyang Chung, Dong Hyun Kim, Kyoung Woo Kim, Jin Kwon Chung, Kyu Yeon Hwang, Ho Sik Hwang, Jin Hyoung Kim, Joon Young Hyon

**Affiliations:** 1 Department of Ophthalmology, Pusan National University Yangsan Hospital, Pusan National University School of Medicine, Yangsan, Republic of Korea; 2 Research Institute for Convergence of Biomedical Science and Technology, Pusan National University Yangsan Hospital, Pusan National University School of Medicine, Yangsan, Republic of Korea; 3 Department of Ophthalmology, Institute of Vision Research, Yonsei University College of Medicine, Seoul, 06273 Republic of Korea; 4 Department of Ophthalmology, Samsung Medical Center, Sungkyunkwan University School of Medicine, Seoul, Republic of Korea; 5 Department of Ophthalmology, Asan Medical Center, University of Ulsan College of Medicine, Seoul, Republic of Korea; 6 Department of Ophthalmology, Kangbuk Samsung Hospital, School of Medicine, Sungkyunkwan University, Seoul, 03181 Republic of Korea; 7 Department of Ophthalmology, College of Medicine, The Catholic University of Korea, Seoul, Republic of Korea; 8 Department of Ophthalmology, Gil Medical Center, Gachon University College of Medicine, Incheon, Republic of Korea; 9 Department of Ophthalmology, Chung-Ang University Hospital, Chung-Ang University College of Medicine, Seoul, Republic of Korea; 10 Department of Ophthalmology, Soonchunhyang University Seoul Hospital, Soonchunhyang University College of Medicine, Seoul, 140-743 Republic of Korea; 11 Department of Ophthalmology, Kim`S Eye Hospital, Seoul, Republic of Korea; 12 Department of Ophthalmology, Yeouido St. Mary’s Hospital, College of Medicine, The Catholic University of Korea, Seoul, Republic of Korea; 13 Department of Ophthalmology, Ilsan Paik Hospital, Inje University College of Medicine, Goyang, Republic of Korea; 14 Department of Ophthalmology, Seoul National University Bundang Hospital, Seoul National University College of Medicine, 82 Gumi-ro 173beon-gil, Bundang-gu, Seongnam-si, Gyeonggi-do Republic of Korea

**Keywords:** Conjunctival diseases, Corneal diseases

## Abstract

Hyaluronic acid (HA) and cyclosporine A (CsA) eyedrops are commonly prescribed in dry eye syndrome (DES). The effectiveness of each preparation in DES is well-known, yet the superiority of one over another has been studied little. We assessed the efficacy and tolerability of 0.15% HA compared to combinations of 0.05% CsA plus 0.5% carboxymethylcellulose (CMC), and 0.15% HA plus 0.05% CsA in patients with moderate to severe DES. Total 438 patients with moderate to severe DES were recruited and randomized for one of the three treatments for 12 weeks. Effectiveness was assessed at baseline, 4- and 12-weeks. The primary endpoint was change in corneal staining score. The secondary endpoints were tear break-up time (TBUT), strip meniscometry (SM) score, ocular surface disease index (OSDI) score, and tolerability questionnaire. The change in corneal staining score for 0.15% HA from the baseline was non-inferior to that of 0.05% CsA. Corneal staining score, TBUT, SM score, and OSDI score improved in all groups without statistically significant intergroup differences. Better tolerability and lower prevalence of adverse drug reactions were seen in 0.15% HA. Our findings suggest that 0.15% HA may be equivalently effective and safer than 0.05% CsA in treating moderate to severe DES.

## Introduction

Dry eye syndrome (DES) is a common, multifactorial disorder associated with evaporation and reduced production of the tear film on the ocular surface^1^. Symptoms of DES include ocular discomfort such as drying, itching, stinging, burning sensation of the eye, redness, and sometimes visual disturbance. These symptoms are usually mild, but occasionally lead to decrease in visual function and reduction in the ability to perform daily visual tasks, reducing the quality of life^2,3^. In addition, DES can make a patient susceptible to corneal infections and can cause mental health disorders such as depression and anxiety^4^. Owing to its high prevalence, 8.5 million people in the US spend more than US$300 million annually on artificial tears and other over-the-counter treatments; the cost of treating DES is increasing with the development of newer treatment options^5^. In this context, research regarding the efficacy and safety of artificial tears for DES is increasing.

Artificial tears are a mainstay treatment for DES. Among the many formulations available, hyaluronic acid (HA) is one of the most commonly prescribed. HA, a natural component of tear film, is a glycosaminoglycan with viscoelastic rheology consisting of repeated disaccharide units of N-acetyl-D-glucosamine and sodium-D-glucuronate^6^. Due to its affinity for water, dehydration resistance, and excellent biocompatibility, it gained widespread application in ocular lubricants^6–8^. Studies have shown that HA reduces the intensity of dry eye symptoms, has a protective effect on corneal epithelial cells against damage, stabilizes the precorneal tear film, stimulates epithelial migration, and improves the optical quality of retinal images^9^.

Recently, it was discovered that in DES, controlling inflammation of the ocular surface is key to improving symptoms, especially in more severe forms of the disease. Cyclosporine A (CsA) is used to treat ocular inflammation. Topical 0.05% CsA is an anti-inflammatory eyedrop with an immunomodulatory mechanism of action which significantly improves corneal and conjunctival staining scores in patients with DES^5,10^. Since it shows no potential side-effects, unlike topical steroids, it is considered to be an effective and reliable long-term treatment option for DES. Additionally, a combination of CsA with carboxymethylcellulose (CMC) is frequently prescribed since it significantly improves the signs and symptoms of DES^11^.

Since both HA and CsA are commonly prescribed eyedrops in DES, a study was conducted to compare their efficacy in DES treatment. Park et al*.* confirmed that 0.1%, 0.15%, and 0.3% HA were as effective as 0.05% CsA in improving the objective signs and subjective symptoms of dry eye^12^. However, to the best of our knowledge, there are no reports that compare the efficacy of 0.15% HA and 0.05% CsA in DES treatment. The present study was designed to evaluate the efficacy of 0.15% HA compared with 0.05% CsA in patients with DES over a 12 week period.

## Methods

The CONSORT checklist for this clinical trial are available in the related files. This study was a multicenter, randomized, investigator-blinded, active-controlled, parallel-group clinical trial in adult patients (≥ 19 years) with DES. The trial was conducted at 15 clinical centers between February 11, 2020 and October 14, 2021. The study protocol was approved by the institutional review board of each institution including Pusan National University Yangsan Hospital Institutional Review Board (No. 02-2019-036, 02/01/2020) and performed in accordance with the Declaration of Helsinki and Good Clinical Practice Guidelines. This trial was registered at the U.S. National Library of Medicine (http://clinicaltrials.gov) (NCT04127851, 16/10/2019).

### Subjects

All patients underwent standard baseline ophthalmic examinations for regular DES. These examinations included a slit-lamp examination, tear break-up time test (TBUT), best-corrected visual acuity (BCVA) test, ocular surface disease index (OSDI) test, corneal fluorescein score examination, and intraocular pressure measurement using a non-contact applanation tonometer. Medical histories were also recorded, including any prior ocular diseases or surgeries, systemic diseases (including diabetes and hypertension), and current medications. Patients with corneal abnormalities or ocular diseases other than DES, history of ocular surgery other than cataract surgery, moderate-to-severe meibomian dysfunction, progression of ocular inflammation, or infectious eye conditions were excluded. The corneal fluorescein score was measured three times in the same eye by one ophthalmologist (1 per site) and the Oxford staining score value was recorded.

### Study protocol

**Figure 1:**
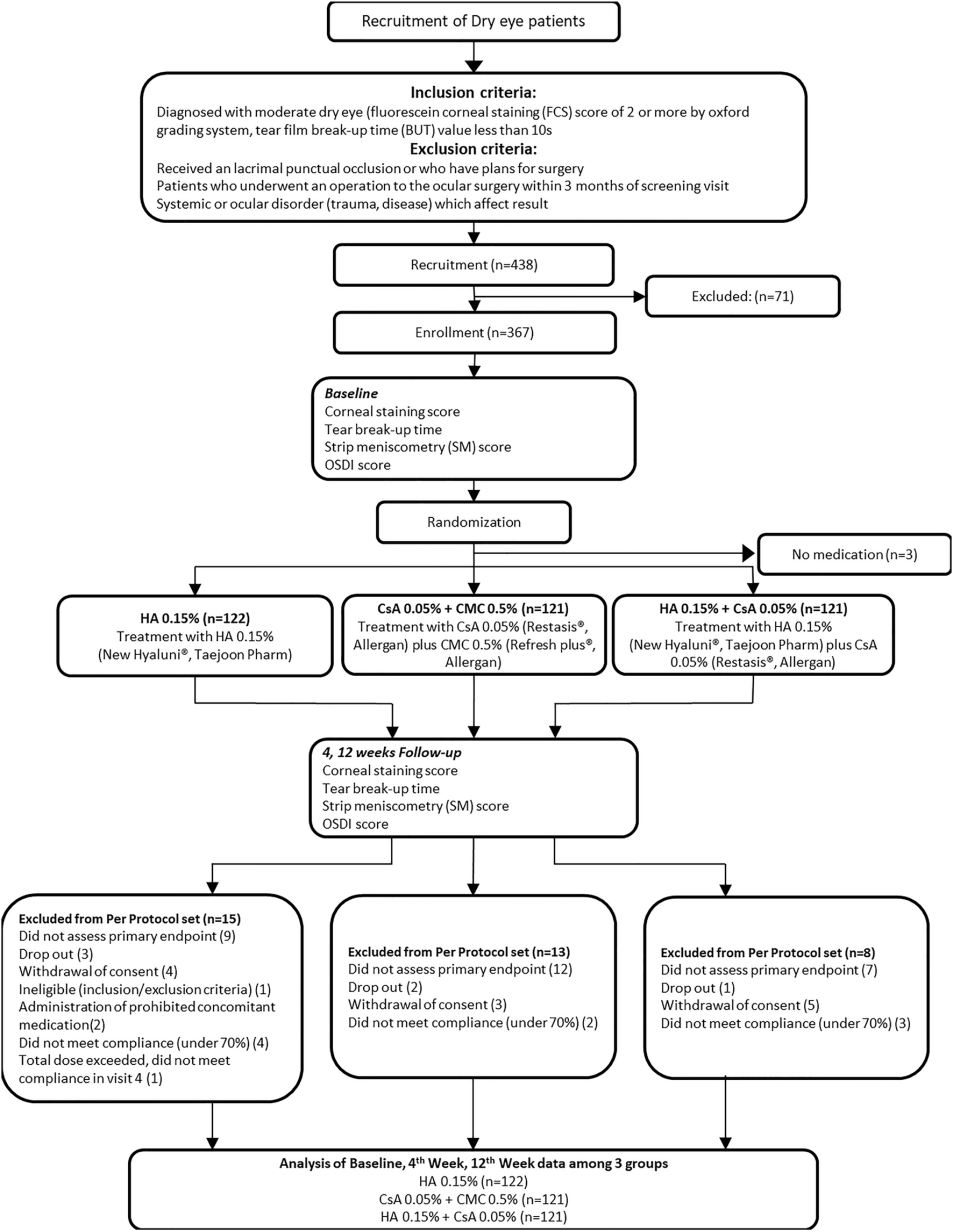
Subject disposition and study protocol described with flowchart.

This study recruited 438 patients, among whom 367 were adults (≥ 19 years of age) with DES (Fig. 1). DES was defined based on the characteristic ocular surface conditions, such as a corneal fluorescein score of more than grade 2 on the Oxford scale or a TBUT of less than 10 s. Patients with DES in eligible eyes (after a washout period) were randomized and assigned to a treatment schedule with daily 0.15% HA (New Hyaluni, Taejoon Pharmaceuticals, Seoul, Korea), a combination of 0.05% CsA (Restasis, Allergan Inc., Irvine, CA) + 0.5% CMC (Refresh plus, Allergan Inc., Irvine, CA, USA), or a combination of 0.15% HA + 0.05% CsA. We excluded patients with 20/80 or lower BCVA on the Snellen chart and those with a medical history of chronic intraocular inflammation within 3 months of screening. We also excluded patients who used contact lenses during the clinical study and those who were pregnant, planning to become pregnant, nursing, or of childbearing potential without a reliable form of contraception. If both eyes met the criteria, the eye with the highest corneal staining score was selected. If the corneal staining scores were equal in both eyes, the right eye was selected. After a washout period of 4 weeks, the subjects were randomized 1:1:1 with 0.15% HA, 0.05% CsA + 0.5% CMC, or 0.15% HA + 0.05% CsA. A total of 367 patients were randomly assigned to each ophthalmic solution group. All patients were scheduled for follow-up visits at weeks 4 and 12.

Patients were divided into three groups based on simple randomization. The 0.15% HA group was treated with 0.15% HA six times daily for 12 weeks. The 0.05% CsA + 0.5% CMC group was treated with 0.05% CsA twice and 0.5% CMC six times daily for 12 weeks. The 0.15% HA + 0.05% CsA group was treated with 0.15% HA six times and 0.05% CsA twice daily for 12 weeks. At the beginning of the treatment period, patients were randomized according to the allocation codes generated for all groups using the permuted restricted block randomization method. Participants were randomized sequentially at each site. All study participants were blinded to treatment assignment for the duration of the study. Only researchers had access to unblinded data.

### Outcome measures

At each follow-up visit, the following evaluations were conducted: corneal staining score, TBUT, strip meniscometry (SM) score, OSDI, and a questionnaire evaluating tolerability. The corneal staining score was evaluated according to the Oxford grading system using fluorescein staining^13^. After applying fluorescein with paper stick, TBUT was evaluated under slit lamp illumination with a cobalt blue light source. We observed the point where black spots, streaks, or fluorescein defects occurred in the tear layer stained with fluorescein after the patient blinked 2–3 times^14^. Time was measured in seconds. The measurement was repeated three times and the average value was used. The OSDI consisted of 12 questions and the total score was calculated by dividing the total score by the number of questions (0 to 4 points for each question). OSDI is expressed as a score ranging from 0 to 100; larger the score, the more severe the symptoms. According to the score distribution, the OSDI was classified as normal (0–12 points), mild (13–22 points), moderate (23–32 points), or severe (33 points or more).

### Statistical analysis

Data are shown as mean ± standard deviation (SD). Monocular data analyses of eligible eyes were performed for statistical comparison. The efficacy analyses consisted of corneal staining score, OSDI score, TBUT, SM score, and tolerability questionnaire at 4 and 12 weeks. Statistical differences between scores of baseline and each week were evaluated using the analysis of covariance and Wilcoxon signed-rank test. A *p*-value (*p*) < 0.05 was considered statistically significant. For the primary endpoint, the non-inferiority of the treatment eyedrop was assessed by calculating the upper limit of the 97.5% one-sided confidence interval for the intergroup difference with a non-inferiority margin of 0.289^15,16^. A sample size of 107 patients per treatment group was planned to provide 80% power to determine non-inferiority based on the mean change in corneal staining score at 12 weeks using a one-sided, two-sample t-test with an alpha of 0.025 and estimated common SD of 0.75^16^. Statistical analyses were performed using SAS v9.2 (SAS Institute, Cary, NC, USA).

## Results

Written informed consent was obtained from all 438 patients recruited for the trial; 367 patients were enrolled and randomized. Among them, three patients did not start medication. Twenty-one patients dropped out; 10 patients did not have the primary endpoint assessed; two patients were administered prohibited concomitant medication; six patients had poor compliance, and 328 patients completed the study without protocol deviation. Thus, there were 328 subjects in the per-protocol set analysis (PPS). The PPS included randomized subjects who took the medication with over 70% compliance and for whom efficacy evaluations were performed within a period of 12 weeks from baseline after medication administration. There was no statistically significant difference between the background patient characteristics with respect to sex and age, regardless of the group.

### Efficacy evaluation

**Figure 2:**
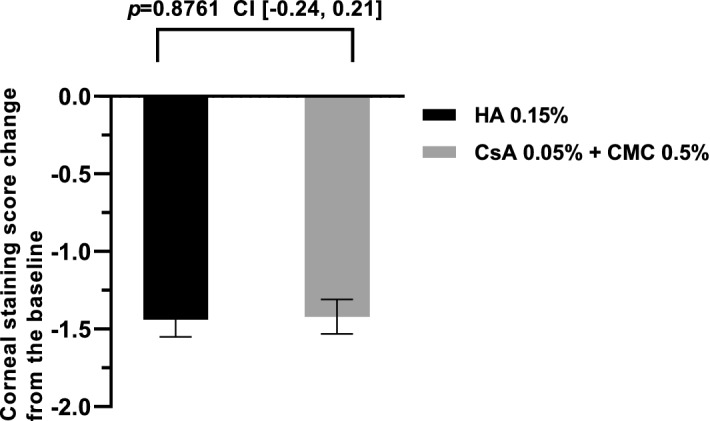
Corneal staining score change from baseline to 12 weeks in each per-protocol set group. Changes in corneal staining score during the follow-up period of 12 weeks in HA 0.15% group and CsA 0.05% + CMC 0.5% group showed no significant difference (Mean value ± standard deviation, *P* > 0.05 by analysis of covariance).

The changes in average corneal staining score were as follows: − 1.44 ± 0.11 in the 0.15% HA group and − 1.42 ± 0.11 in the 0.05% CsA + 0.5% CMC group (Fig. 2). We confirmed non-inferiority in the changes of corneal staining score of the 0.15% HA group compared to the 0.05% CsA + 0.5% CMC group at 12 weeks [95% CI (− 0.24, 0.21), *P* = 0.8761]. The difference of upper limit of the confidence interval between the 0.15% HA group and 0.05% CsA + 0.5% CMC group was 0.21 points, which was less than the non-inferiority margin of 0.289, confirming the non-inferiority of the 0.15% HA group.

#### Corneal staining score

**Figure 3:**
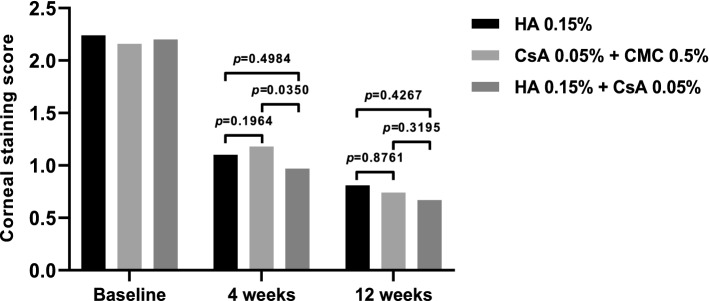
Corneal staining score at 4 and 12 weeks in each per-protocol set group. Corneal staining scores of 0.05% CsA + 0.5% CMC and 0.15% HA + 0.05% CsA show significant difference on 4 weeks. (*P* < 0.05 by analysis of covariance). On 12 weeks all groups showed no significant intergroup difference (Mean value ± standard deviation, *P* > 0.05 by analysis of covariance).

The corneal staining scores of all treatment groups decreased significantly from baseline at all visits (Fig. 3). At the first visit after initiation of instillation (4 weeks), there was a statistically significant difference in corneal staining scores between the 0.05% CsA + 0.5% CMC and 0.15% HA + 0.05% CsA group (*P* = 0.0350). However, there was no statistically significant intergroup difference at either 4 or 12 weeks. The average corneal staining scores were as follows: 2.24 ± 0.51 at baseline, 1.10 ± 0.91 at 4 weeks, and 0.81 ± 0.90 at 12 weeks in the 0.15% HA group; 2.16 ± 0.37 at baseline, 1.18 ± 0.89 at 4 weeks, and 0.74 ± 0.81 at 12 weeks in the 0.05% CsA + 0.5% CMC group; 2.20 ± 0.47 at baseline, 0.97 ± 0.81 at 4 weeks, and 0.67 ± 0.73 at 12 weeks in the 0.15% HA + 0.05% CsA group.

#### TBUT

**Figure 4:**
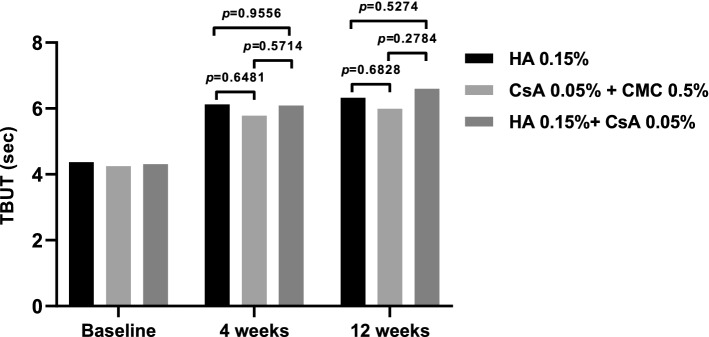
TBUT at 4 and 12 weeks in each per-protocol set group. **T**BUT on each follow-up period showed no significant intergroup difference (Mean value ± standard deviation, *P* > 0.05 by analysis of covariance).

TBUT in all treatment groups increased significantly from the baseline at all subsequent visits. No significant differences were observed between the groups at either visit (Fig. 4). The average TBUT was as follows: 4.37 ± 1.88 at baseline, 6.12 ± 3.62 at 4 weeks, and 6.32 ± 3.73 at 12 weeks in the 0.15% HA group; 4.25 ± 1.61 at baseline, 5.78 ± 3.35 at 4 weeks, and 5.99 ± 3.61 at 12 weeks in the 0.05% CsA + 0.5% CMC group; 4.31 ± 1.68 at baseline, 6.09 ± 3.39 at 4 weeks, and 6.60 ± 3.78 at 12 weeks in the 0.15% HA + 0.05% CsA group.

#### SM score

**Figure 5:**
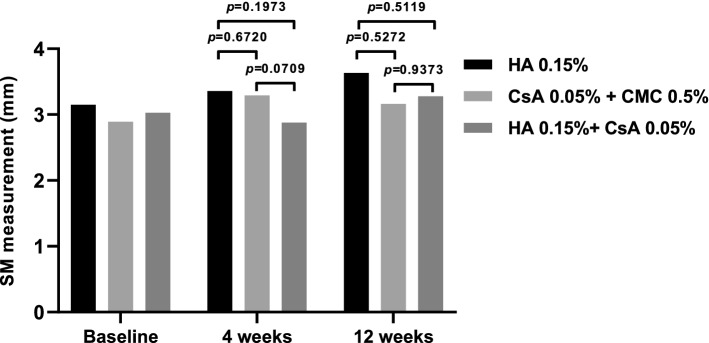
Strip meniscometry (SM) scores at 4 and 12 weeks in each per-protocol set group. SM scores on each follow-up period showed no significant intergroup difference (Mean value ± standard deviation, *P* > 0.05 by analysis of covariance).

SM scores in all treatment groups increased slightly from baseline at all subsequent visits. No significant differences were observed between the groups at either visit (Fig. 5). The average SM scores were as follows: 3.15 ± 1.80 at baseline, 3.36 ± 2.59 at 4 weeks, and 3.63 ± 2.43 at 12 weeks in the 0.15% HA group; 2.89 ± 1.87 at baseline, 3.29 ± 1.95 at 4 weeks, and 3.16 ± 1.92 at 12 weeks in the 0.05% CsA + 0.5% CMC group; 3.03 ± 1.78 at baseline, 2.88 ± 1.56 at 4 weeks, and 3.28 ± 2.22 at 12 weeks in the 0.15% HA + 0.05% CsA group.

#### OSDI score

**Figure 6:**
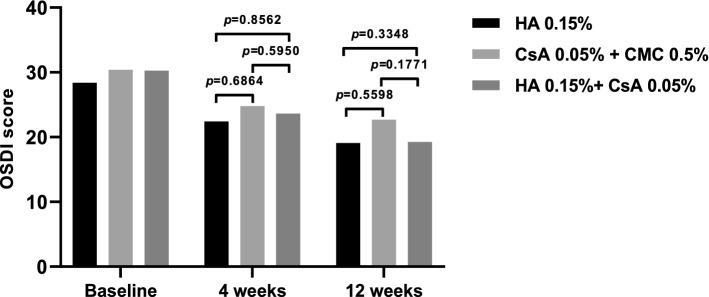
Ocular surface disease index (OSDI) scores at 4 and 12 weeks of the per-protocol set group. OSDI scores on each follow-up period showed no significant intergroup difference (Mean value ± standard deviation, *P* > 0.05 by analysis of covariance).

The OSDI scores in all treatment groups decreased significantly from baseline at all later visits (Fig. 4). There were no significant differences between the groups at each visit (Fig. 6). The average OSDI scores were as follows: 28.39 ± 18.09 at baseline, 22.42 ± 16.71 at 4 weeks, and 19.09 ± 14.73 at 12 weeks in the 0.15% HA group; 30.39 ± 17.30 at baseline, 24.77 ± 14.20 at 4 weeks, and 22.67 ± 14.93 at 12 weeks in the 0.05% CsA + 0.5% CMC group; 30.26 ± 19.46 at baseline, 23.61 ± 15.05 at 4 weeks, and 19.26 ± 13.22 at 12 weeks in the 0.15% HA + 0.05% CsA group.

### Tolerability and safety

With regards to tolerability, this study showed that the prevalence of ‘stinging/burning’, ‘sandiness/grittiness’, ‘light sensitivity’, and ‘pain or soreness’ in the 0.15% HA group were significantly less, compared to the 0.05% CsA + 0.5% CMC group (*P* < 0.0001 at 4 weeks and 12 weeks; *P* = 0.0336 at 4 weeks and *P* = 0.0017 at 12 weeks; *P* = 0.0355 at 4 weeks and *P* = 0.0285 at 12 weeks; *P* = 0.0024 at 4 weeks and *P* = 0.0016 at 12 weeks, respectively). There was no statistically significant difference in ‘blurred vision’, ‘dryness’, or ‘foreign body sensation’ (*P* > 0.05 at 4 weeks and 12 weeks) (Table 1).

**Table 1:** Tolerability evaluation at baseline, 4, and 12 weeks in the safety set group.

	Symptom score (mean ± SD) (%)				
	0.15% HA	0.05% CsA + 0.5% CMC		0.15% HA + 0.05% CsA	
			*P* value^a^		*P* value^b^
**Stinging/burning**					
Baseline	0.43 ± 0.70	0.40 ± 0.68		0.35 ± 0.68	
4 weeks	0.47 ± 0.66	1.10 ± 0.93	< 0.0001	0.94 ± 0.96	< 0.0001
12 weeks	0.39 ± 0.69	0.87 ± 0.86	0.0002	0.69 ± 0.89	0.0060
**Sticky eye sensation**					
Baseline	0.20 ± 0.50	0.23 ± 0.54		0.17 ± 0.44	
4 weeks	0.36 ± 0.65	0.45 ± 0.65	0.3221	0.28 ± 0.57	0.4309
12 weeks	0.28 ± 0.56	0.40 ± 0.67	0.1760	0.25 ± 0.52	0.7357
**Itching**					
Baseline	0.39 ± 0.76	0.35 ± 0.67		0.30 ± 0.67	
4 weeks	0.47 ± 0.76	0.34 ± 0.63	0.3858	0.33 ± 0.65	0.7635
12 weeks	0.38 ± 0.67	0.35 ± 0.64	0.8181	0.28 ± 0.54	0.8918
**Blurred vision**					
Baseline	0.57 ± 0.84	0.58 ± 0.81		0.45 ± 0.81	
4 weeks	0.73 ± 0.88	0.86 ± 0.86	0.3045	0.54 ± 0.78	0.8006
12 weeks	0.61 ± 0.82	0.79 ± 0.72	0.0326	0.50 ± 0.71	0.4671
**Sandiness/Grittiness**					
Baseline	0.47 ± 0.79	0.38 ± 0.71		0.40 ± 0.73	
4 weeks	0.50 ± 0.71	0.61 ± 0.80	0.1320	0.55 ± 0.81	0.3482
12 weeks	0.44 ± 0.72	0.49 ± 0.72	0.1766	0.40 ± 0.63	0.4453
**Dryness**					
Baseline	0.84 ± 1.04	0.81 ± 1.04		0.63 ± 0.95	
4 weeks	0.67 ± 0.88	0.68 ± 0.89	0.5843	0.55 ± 0.89	0.3616
12 weeks	0.67 ± 0.91	0.60 ± 0.82	0.9127	0.51 ± 0.73	0.3689
**Light sensitivity**					
Baseline	0.37 ± 0.69	0.42 ± 0.79		0.37 ± 0.75	
4 weeks	0.35 ± 0.68	0.36 ± 0.71	0.6615	0.33 ± 0.63	0.6518
12 weeks	0.30 ± 0.60	0.45 ± 0.77	0.2620	0.26 ± 0.52	0.7952
**Pain or soreness**					
Baseline	0.22 ± 0.58	0.24 ± 0.52		0.16 ± 0.43	
4 weeks	0.26 ± 0.55	0.58 ± 0.79	0.0008	0.53 ± 0.78	0.0002
12 weeks	0.20 ± 0.50	0.43 ± 0.73	0.0394	0.41 ± 0.71	0.0062

A score of 0 = no symptoms, a score of 4 = always had symptoms.

*SD* standard deviation, *HA* hyaluronic acid, *CsA* cyclosporine A, *CMC* carboxymethylcellulose.

^a^Difference between the 0.15% HA and 0.05% CsA + 0.5% CMC group (Wilcoxon rank sum test).

^b^Difference between the 0.15% HA and 0.15% + 0.05% CsA group (Wilcoxon rank sum test).

The adverse events that occurred during the study period are summarized in Table 2. A total of 96 adverse events developed in 70 of the 364 patients who received investigational drugs at least once. Thirty cases occurred in 24 patients in the 0.15% HA group, 34 cases in 24 patients in the 0.05% CsA + 0.5% CMC group, and 33 cases in 22 patients in the 0.15% HA + 0.05% CsA group. There was no statistically significant difference in the incidence of adverse events in the 0.15% HA group compared to those in the 0.05% CsA + 0.5% CMC and 0.15% HA + 0.05% CsA groups (*P* = 0.9746 and 0.7432, respectively). In contrast, there was a statistically significant difference in the incidence of adverse drug reactions between the 0.15% HA group and the 0.05% CsA + 0.5% CMC and 0.15% HA + 0.05% CsA group (*P* = 0.0225 and 0.0225, respectively). In the 0.15% HA group, there was one case of ‘eye pain’, ‘conjunctivitis’, ‘allergy’, and ‘foreign body sensation in the eyes’. In the 0.05% CsA + 0.5% CMC group, there were 10 cases of ‘eye pain’, two cases of ‘ocular hyperemia’, and one case of ‘conjunctival hemorrhage’. In the 0.15% HA + 0.05% CsA group, there were 4 cases of ‘eye pain’, three cases of ‘eye irritation’, and one case each of ‘abnormal sensation in the eye’, ‘asthenopia’, ’blepharitis’, ‘eye discharge’, ‘eye pruritus’, ‘ocular discomfort’, and ‘visual impairment’ (Table 3).

**Table 2:** Adverse events evaluated in the safety set.

Safety profile	HA 0.15%	CsA 0.05% + CMC 0.5%	HA 0.15% + CsA 0.05%	*P* value^a^	*P* value^b^	*P* value^c^
Adverse event (AE)	30	34 24(19.83)	33	0.9746	0.7668	0.7432
Number of subjects (%)	24(19.67)		22 (18.18)			
Adverse drug reaction (ADR) Number of subjects (%)	4	17	15	0.0225	0.0225	1.0000
	4 (3.28)	13 (10.74)	13 (10.74)			
Serious AE	1	0	0	1.0000	1.0000	NA
Number of subjects (%)	1 (0.82)	0 (0.0)	0 (0.0)			
Serious ADR	0	0	0	NA	NA	NA
Number of participants (%)	0 (0.0)	0 (0.0)	0 (0.0)			
AEs resulting in drop out	0	0	1	NA	0.4979	1.0000
Number of subjects (%)	0 (0.0)	0 (0.0)	1 (0.83)			

*HA* hyaluronic acid, *CsA* cyclosporine A, *CMC* carboxymethylcellulose.

^a^Difference between 0.15% HA and 0.05% CsA + 0.5% CMC group.

^b^Difference between 0.15% HA and 0.15% HA + 0.05% CsA group.

^c^Difference between 0.05% CsA and 0.15% HA + 0.05% CsA group.

**Table 3:** Eye disorders among adverse drug reactions.

Safety profile	HA 0.15%	CsA 0.05% + CMC 0.5%	HA 0.15% + CsA 0.05%
Subjects with eye disorder (%)	3 (2.46)	12 (9.92)	12 (9.92)
Eye pain	1 (0.82)	10 (8.26)	4 (3.31)
Eye irritation	0	1 (0.83)	2 (1.65)
Ocular hyperemia	0	2 (1.65)	0
Abnormal sensation in eye	0	0	1 (0.83)
Asthenopia	0	0	1 (0.83)
Blepharitis	0	0	1 (0.83)
Conjunctival hemorrhage	0	1 (0.83)	0
Conjunctivitis allergic	1 (0.82)	0	0
Eye discharge	0	0	1 (0.83)
Eye pruritus	0	0	1 (0.83)
Foreign body sensation in eyes	1 (0.82)	0	0
Ocular discomfort	0	0	1 (0.83)
Visual impairment	0	0	1 (0.83)

*HA* hyaluronic acid, *CsA* cyclosporine A, *CMC* carboxymethylcellulose.

## Discussion

The present study was designed to prove the non-inferiority of 0.15% HA compared to the combination of 0.05% CsA + 0.5% CMC with respect to clinical efficacy, in terms of changes in ocular staining score as a primary endpoint from baseline to 12 weeks. Additionally, by adding the 0.15% HA + 0.05% CsA group, we examined whether HA had a synergistic effect with CsA. We found that the corneal staining score, TBUT, SM score, and OSDI score during 12 weeks of the follow-up period were not significantly different between the three groups. In addition, as our primary outcome, we confirmed that the change in corneal staining score of the 0.15% HA group was statistically non-inferior to that of the 0.05% CsA + 0.5% CMC group. Furthermore, we found that the corneal staining score of the 0.15% HA + 0.05% CsA group at 4 weeks was significantly lower than that of the 0.05% CsA + 0.5% CMC group.

DES, which accompanies tear deficiency and changes tear properties, often results in corneal epithelial barrier dysfunction and superficial epithelial lesions^17^. Thus, changes in corneal staining score during the treatment period with artificial tears could quantitatively effect ocular surface integrity. The results of our study showed a similar decrease in corneal staining score at all visits, implying that both 0.15% HA and 0.05% CsA + 0.5% CMC group comparably improved the health of the ocular surface.

Tear film stability is an important aspect of tear film changes that occur in DES as it is linked to the health of the ocular surface^18^. TBUT is commonly used to evaluate the effect of artificial tears. The measurement of TBUT in this study was done with slit-lamp examination using fluorescein dye, though there is some controversy in its reproducibility and objectivity^19,20^. Nevertheless, the results of this study demonstrated that TBUT significantly decreased in the three groups at all visits, while showing no significant differences among the groups. The SM score evaluates the tear meniscus volume of the participants. Although it has a similar controversy as TBUT, it has been reported to have good reproducibility and repeatability and is correlated with TBUT, Schirmer’s I test, and tear meniscus height in DES^21^. In terms of SM score, there were similar improvements in all groups, with no significant differences among them. These results suggest that all three treatments similarly improved both the integrity of the tear film and the tear volume, inhibiting the evaporation of the tear film and maintaining its osmolarity^22^. Additionally, we assessed the subjective efficacy of artificial tears using the OSDI score. The OSDI is a 12-item questionnaire about ocular irritation symptoms which helps in the subjective evaluation of DES^23^. Studies reported that the subjective ocular discomfort might be in positive correlation with tear osmolarity, which is the only objective parameter in diagnosing DES^24,25^. Similar to other parameters, all three groups showed a tendency for improvement without significant intergroup differences.

HA has been reported to have various mechanisms of action in DES treatment. First, HA has wound healing properties on the corneal epithelium^26^. Several in vitro studies have demonstrated that HA inactivates the CD44 adhesion molecule, which is overexpressed in the cornea and conjunctiva of eyes with DES^27^. HA, as a ligand, is thought to bind with CD44, inducing cell-to-cell interactions to stabilize corneal epithelium adhesion to the basement membrane, promoting cellular migration and re-epithelization^19,28,29^. Second, HA can reduce mechanical damage to the cornea because it has highly viscoelastic properties that lubricate the ocular surface during blinking and ocular movements, preventing frictional damage from the eyelid^30^. Third, HA may play a role in the regulation of localized inflammation in patients with keratoconjunctivitis sicca^31^. In addition, since HA has significant water-retentive properties with an affinity of 1000-fold its own weight, it assists the ocular surface in resisting dehydration, reinforcing wettability, and reducing evaporation of the tear film^19^. HA could also help in the natural healing of keratitis at an early stage by acting as a protective coating over the wound, thus preventing further irritation and damage to the cornea^28^.

Topical 0.05% CsA is the first and only FDA-approved therapeutic artificial tear for the treatment of chronic DES. It modulates the underlying inflammatory pathology of the ocular surface by increasing tear production. It is a type of calcineurin inhibitor, similar to tacrolimus and voclosporin. CsA binds to cyclophilin in lymphocytes and inhibits calcineurin, preventing expression of immune mediators, such as interleukin (IL)-2, IL-4, and interferon-γ^5^. As IL-2 is essential for T-cell replication, Cs is a potent inhibitor of T-cell proliferation and thereby inhibits T cell-mediated immune responses. Studies have shown that CsA exerts a marked therapeutic effect on patients with dry eye by inhibiting T lymphocytes. In DES, environmental and physiological factors disturb the homeostatic system of the ocular surface, creating an imbalance between secretion and degradation of the components of the tear film, poor tear secretion and clearance, or changes in tear composition. This instability increases susceptibility to desiccation and damage to the corneal epithelium, leading to the release of inflammatory mediators. Throughout the inflammatory response, immune cells release pro-inflammatory cytokines and chemokines, which recruit more immune cells, resulting in a vicious cycle of inflammation that does not resolve. CsA interrupts this cycle by blocking T cell infiltration, activation, and the subsequent release of inflammatory cytokines^32^. Moreover, CsA protects human conjunctival epithelial cells via its anti-apoptotic action and improves conjunctival goblet cell density, providing a healthy ocular surface^33,34^. CsA may also prevent opening of the mitochondrial permeability transition pore with effects of inhibition of cytochrome c release and a decrease in cell apoptosis^5^. Gürdal et al*.* showed that topical 0.05% CsA inhibits both apoptosis and matrix metalloproteinase-9 expression in the conjunctival epithelial cells of patients with DES^32^.

Both HA and CsA have been reported to be effective in DES. As HA is widely used for mild DES, many studies have confirmed its efficacy in both the subjective and objective aspects of DES. Johnson et al*.* reported that 0.1% and 0.3% HA improved non-invasive TBUT in a short-term follow-up of 1 week when compared to 0.9% saline eyedrops^6^. Brignole et al*.* reported that HA improved corneal staining score at 8 weeks of follow-up and showed efficacy faster than CMC, implying an advantage for early DES patients^28^. Lee et al*.* also reported, in an 8 week study period, that HA significantly improved corneal and conjunctival staining scores, TBUT, and subjective symptoms of DES^35^. Aragona et al. confirmed the efficacy of HA over a longer period of 12 weeks by grading epithelial cytology^30^. In a meta-analysis by Yang et al*.,* HA administration led to superior improvements in Schirmer’s I test with similar results in TBUT compared to non-HA type artificial tears. There have been few study comparing the effect of different concentration of HA on DES patients, but in the study of Park et al., 0.1%, 0.15%, and 0.3% HA showed no intergroup difference in corneal staining score, TBUT, Schirmer's I test score, OSDI score, and the prevalence of adverse effects in DES patients^12^. On the other hand, Sall et al*.* showed that treatment with CsA resulted in significant improvements in corneal staining score, Schirmer's I test score, and subjective measure of DES in a 6-month follow-up period^10^. In a meta-analysis of 1660 patients in 12 randomized trials, topical CsA significantly improved both TBUT and Schirmer's I test score, and could be more effective for DES patients with conjunctival injuries^36^. However, because of its nature as an anti-inflammatory agent, the Delphi panel in 2006 proposed for CsA to only be used in moderate to severe DES, when there are signs of clinical inflammation^37^.

In this context, few studies have compared HA and CsA. Only one clinical study by Park et al. compared the efficacy between the two agents and concluded that 0.1%, 0.15%, and 0.3% HA, and 0.05% CsA improved corneal staining score, TBUT, and OSDI score, without significant intergroup statistical differences^12^. Similarly, in the present study, both 0.15% HA and 0.05% CsA therapy for 12 weeks showed a comparable effect on corneal staining score, TBUT, SM score, and OSDI score. However, in the 0.05% CsA + 0.5% CMC group, because the subjects were also treated with 0.5% CMC, it was not possible to exclude its additional effect. Thus, the efficacy of 0.15% HA may be higher than that of 0.05% CsA. Additionally, the synergistic effect of 0.15% HA and 0.05% CsA was not statistically significant, confirming the clinical usefulness of 0.15% HA monotherapy. This result seems to imply that protection of the corneal epithelium should be more important than modulating the inflammatory response for treatment of moderate to severe DES. Further studies should be conducted to clarify the exact mechanisms underlying these results. In addition, the significant decrease in corneal staining score of the 0.15% HA + 0.05% CsA group at 4 weeks, compared to the 0.05% CsA + 0.5% CMC group, might reflect the synergistic effect of 0.15% HA + 0.05% CsA treatment; this treatment exerts its efficacy in a shorter period of time in DES patients.

Both 0.15% HA and 0.05% CsA were generally well tolerated. However, our results showed the prevalence of ‘stinging/burning’, ‘sandiness/grittiness’, ‘light sensitivity’, and ‘pain or soreness’ symptoms in the 0.15% HA group were significantly less, when compared to the 0.05% CsA + 0.5% CMC group. The types and prevalence of adverse events (AEs) among the groups were similar with no statistically significant intergroup differences. There were 0 or just 1 ‘serious AE’ or ‘AE resulting in drop-out’ reported in each group. However, in terms of adverse drug reactions (ADRs), the 0.15% HA groups showed significantly lower prevalence than both the 0.05% CsA + 0.5% CMC and 0.15% HA + 0.05% CsA groups. In particular, the 0.05% CsA + 0.5% CMC group showed a relatively high frequency of eye pain related to the instillation of the eye solution compared to the 0.15% HA group. This result is consistent with previous clinical trials, which reported 15–25% of eyes instilled with 0.05% CsA had ‘burning’ or ‘stinging’ eye pains^10,36^. The relatively fewer cases of drug related eye disorders in the 0.05% CsA + 0.5% CMC group (9.92%) versus previous studies could be attributed to concomitant 0.5% CMC instillation.

This study had some limitations. First, the difference in instillation frequency among the groups could have influenced the outcomes. However, considering that 0.05% CsA was instilled with 0.5% CMC six times daily, the higher instillation frequency of the 0.15% HA group could be offset. Second, the total follow-up period of 12 weeks was relatively short, and it might be insufficient to compare the impact of CsA on chronic DES since CsA is believed to affect subconjunctival inflammation by inhibiting T cells, which may take 4–6 months of treatment for relief^38^. However, CsA eyedrops were shown to improve objective measures of dry eye, such as TBUT and Schirmer’s I test, in a 6–8 week follow up period^39^. Furthermore, improvements in all DES parameters from 4 to 12 weeks were less than those from baseline to 4 weeks in the 0.05% CsA + 0.5% CMC group. This indicates that the loading effect of CsA did not appear in our results; this could be due to the effect of 0.5% CMC, which was instilled with 0.05% CsA.

In conclusion, treatment with 0.15% HA seems to be as effective as 0.05% CsA + 0.5% CMC or 0.15% HA + 0.05% CsA for patients with moderate to severe DES in short-term period, improving both objective and subjective aspects of DES. 0.15% HA patients showed better tolerability results for ‘stinging/burning’, ‘sandiness/grittiness’, ‘light sensitivity’, and ‘pain or soreness’. Additionally, 0.15% HA had a non-inferior prevalence of AEs and showed significantly fewer ADRs when compared to the 0.05% CsA + 0.5% CMC and 0.15% HA + 0.05% CsA groups. Considering the effects of the 0.5% CMC used in the 0.05% CsA + 0.5% CMC groups, treating DES patients with the daily use of 0.15% HA may be equivalent and more tolerable than 0.05% CsA.

## Supplementary Information

Supplementary Information.

## Data Availability

The datasets generated and analyzed in the current study are available from the corresponding author on reasonable request.
