# Supplementary material for: A randomized multicenter evaluation of the efficacy of 0.15% hyaluronic acid versus 0.05% cyclosporine A in dry eye syndrome
Source: Sci Rep. 2022 Nov 4;12:18737. doi: 10.1038/s41598-022-21330-0 (PMC9636392; doi:10.1038/s41598-022-21330-0)
Supplement: Supplementary file 1 — Supplementary Information. [file 41598_2022_21330_MOESM1_ESM.pdf]

A Multicenter, Randomized, Single-blind Evaluation of the Efficacy of 0.15% Hyaluronic Acid versus 0.05% Cyclosporine A in Moderate to Severe Dry Eye Syndrome

Ji Eun Lee[1,2†], Sangyoon Kim[1,2†], Hyung Keun Lee[3], Tae-Young Chung[4], Jae Yong Kim[5], Chul Young Choi[6], So Hyang Chung[7], Dong Hyun Kim[8], Kyoung Woo Kim[9], Jin Kwon Chung[10], Gyu Yeon Hwang[11], Ho Sik Hwang[12], Jin Hyung Kim[13] , Joon Young Hyon[14]*

[1]Department of Ophthalmology, Pusan National University Yangsan Hospital, Pusan National University School of Medicine, Yangsan, Korea

[2]Research Institute for Convergence of Biomedical Science and Technology, Pusan National University Yangsan Hospital, Pusan National University School of Medicine, Yangsan, Korea

[3]Institute of Vision Research, Department of Ophthalmology, Yonsei University College of Medicine, Seoul 06273, Korea.

[4]Department of Ophthalmology, Samsung Medical Center, Sungkyunkwan University School of Medicine, Seoul, Korea

[5]Department of Ophthalmology, Asan Medical Center, University of Ulsan College of Medicine, Seoul, Republic of Korea

[6]Department of Ophthalmology, Kangbuk Samsung Hospital, School of Medicine, Sungkyunkwan University,Seoul 03181, Korea

[7]Department of Ophthalmology, The Catholic University of Korea Seoul St. Mary's Hospital, Seoul, Korea

[8]Department of Ophthalmology, Gil Medical Center, Gachon University College of Medicine, Incheon, Korea.

[9]Department of Ophthalmology, Chung-Ang University Hospital, Chung-Ang University College of Medicine, Seoul, Korea

[10]Department of Ophthalmology, Soonchunhyang University College of Medicine, Soonchunhyang University Seoul Hospital, Seoul 140-743, Republic of Korea

[11]Department of Ophthalmology,KIM`s Eye Hosptial, Seoul, Korea

[12]Department of Ophthalmology, Yeouido St. Mary's Hospital, College of Medicine, The Catholic University of Korea, Seoul

[13]Department of Ophthalmology, Paik Hospital, Ilsan, Inje Medical College, Korea.

[14]Department of Ophthalmology, Seoul National University Bundang Hospital, Seoul National University College of Medicine, Seongnam, Republic of Korea

†These authors contributed equally to this work.

*Corresponding author:

Joon Young Hyon, MD, PhD

Department of Ophthalmology, Seoul National University Bundang Hospital, Seongnam, Korea

82 Gumi-ro 173beon-gil, Bundang-gu, Seongnam-si, Gyeonggi-do, Korea

TEL: +82-31-787-7379

FAX: +82-31-787-4057

E-mail: jyhyon@gmail.com

SUPPLEMENTARY TABLES

Supplementary table S1. The number of right eyes included in each group

	HA 0.15%	CsA 0.05%+CMC 0.5%	HA 0.15%+CsA 0.05%
**Right eye, number (%)**	81 (66.39)	86 (71.07)	75 (61.98)

Supplementary table S2. Baseline severity proportion of OSDI score. There is no statistical difference between groups (p>0.05)

	HA 0.15%	CsA 0.05%+CMC 0.5%	HA 0.15%+CsA 0.05%
**Under 13, number (%)**	25 (20.49)	27 (22.31)	26 (22.31)
**Mild(13~22), number (%)**	27 (22.13)	24 (19.83)	29 (23.77)
**Moderate(23~32), number (%)**	26 (21.31)	21 (17.35)	26 (21.31)
**Severe(33~100), number (%)**	44 (36.06)	49 (40.49)	41 (33.60)

Supplementary table S3. Baseline severity proportion of eyes with corneal staining. There is no statistical difference between groups (p>0.05)

	HA 0.15%	CsA 0.05%+CMC 0.5%	HA 0.15%+CsA 0.05%
**Grade 1, number (%)**	-	-	-
**Grade 2, number (%)**	97(79.50)	102(84.29)	101(83.47)
**Grade 3, number (%)**	20(16.39)	19(15.70)	18(14.75)
**Grade 4, number (%)**	5 (4.09)	-	3 (2.45)
